# Evaluation of a positioning method for equine lateral stifle scintigrams

**DOI:** 10.1186/1751-0147-54-38

**Published:** 2012-06-15

**Authors:** Marion Grapperon Mathis, Charles Ley, Mieth Berger, Kerstin Hansson

**Affiliations:** 1 Section of Diagnostic Imaging, University Animal Hospital Swedish University of Agricultural Sciences, Uppsala, Sweden; 2 Department of Clinical Sciences, Swedish University of Agricultural Sciences, Uppsala, Sweden

## Abstract

**Background:**

The current lack of a standardized protocol for positioning of the gamma camera relative to the horse limb in a lateral stifle scintigram, and thus the reliance on subjective positioning, may be a cause of diagnostic error and inter-operator variability due to variations of the view angle. The aims of this study were to develop a reliable method to obtain a lateral scintigram of the equine stifle based on fixed anatomical landmarks and measure the resulting foot to gamma camera angle on sequential measurements of the same horse and of different horses

**Methods:**

Technetium filled capsules were glued on the skin on sites adjacent to the origin of the medial and lateral femorotibial collateral ligaments in 22 horses using ultrasound guidance. A lateral view of the stifle was defined as the image where the two radioactive point sources were aligned vertically (point sources guided method). Five sequential lateral acquisitions (one to five) of the stifle with the point sources vertically aligned were acquired in each horse, and the angle between the mid-sagittal foot-axis and the vertical axis of the gamma camera (FC angle) was measured for each of these acquisitions

**Results:**

For acquisition group one to five, the mean of the means FC angle was 91.6 ± 2° (2SD) and the coefficient of variation (COV) was 1.1%. In the 22 horses the 95% CI for the mean FC angles was 91.6° ± 12.1° (2SD) and the COV was 6.6%.

**Conclusions:**

The use of point sources to guide gamma camera position results in less variation in the lateral scintigram than if the distal limb is used as guidance due to a difference in FC angle between horses. The point source guided positioning method is considered suitable as a reference standard method to obtain lateral scintigrams of the equine stifle, and it will be of value in clinical scintigraphy and research. The use of alignment of specifically located point sources may also be applied in other regions to standardize scintigraphic views.

## Background

Nuclear scintigraphy is a well-established technique for the investigation of lameness in equine practice. Bone-phase images obtained using diphosphonate radiopharmaceuticals give images that reflect osteoblast activity and regional blood flow to the skeleton, thus an image of function rather than morphology [1,2]. The distribution of the radiopharmaceutical uptake (RU) in the tissues is represented by the distribution of counts per pixel in the scintigram. A region with high RU will be seen as a region with high counts per pixel or high intensity, and any region with RU differing from the normal variations will be classified as abnormal.

Scintigrams can be assessed both subjectively and objectively. The subjective evaluation relies on the knowledge of the normal variations of the RU pattern expected in a specific anatomical region. Radiopharmaceutical uptake is subjectively classified according to its intensity, location and distribution pattern. Objective evaluation of scintigrams is based on the number of counts per pixel in a specifically defined region of interest (ROI). When the counts per pixel of ROIs are compared through the use of ratios, the evaluation is called semi-quantitative. Since a ROI only examines a small part of the image then precise anatomical placement of the ROI is essential to avoid misinterpretations and accuracy can be achieved only if reliable scintigraphic views are obtained.

The two-dimensional nature of scintigraphy means that accurate comparisons of uptake patterns of an anatomical region in scintigrams require a constant angle of the gamma camera to the anatomy. The same concept applies in radiology [3], although radiology has the advantage that the relative position of anatomical structures in the radiograph can be assessed with greater detail. Positioning error is one of the most common reasons for equine radiographs being considered non-diagnostic, and it is suggested that the most common reason for positioning errors of the latero-medial stifle radiographs is due to the radiographic beam being aligned perpendicular to the horse and not the limb [4]. Due to the larger size and curved shape of the stifle, the anatomical landmarks may be more difficult to define compared to the distal limb. The relatively poor anatomical information provided by a scintigram means that even relatively large variations in view angle might not be obvious when images are evaluated.

The authors of a recent review paper [1] concluded that despite a growing literature on the indications for scintigraphy in the horse, in the majority of published studies, inherent biases in study design make it difficult to accurately assess the validity of skeletal scintigraphy. A commonly cited bias is the use of a reference standard that is not accurate [1]. The most commonly mentioned view for stifle scintigraphy in standing horses is the lateral view. However, to the authors’ knowledge a standardized positioning method to obtain a lateral view of the equine stifle is not described in the literature. Currently, the positioning of the gamma camera relative to the horse limb is subjective and the lack of standardized protocol may be a potential source of diagnostic error and inter-observer variability due to variations of the view angle [5]. Phantom models are useful for investigating position of anatomical landmarks and intensity in scintigrams [6-8], but currently there is no such model available for lateral scintigrams of the equine stifle.

The lateral view can be complemented with a caudal view, but caudal views commonly have poor anatomical resolution and lesion definition due to the relatively thick soft tissue covering of the skeleton in this view [9]. If orthogonal views for the lateral view are not available or if image quality is poor, then anatomical localization of abnormal RU in a lateral plane can be uncertain. In any event, it is important to have a consistent lateral view angle, since oblique angles will result in change of the spatial relationships of RUs in the scintigram. Moreover, the tissue thickness in this region attenuates greatly the emitted radiation and incorrect position may impair even more the detection of certain RUs.

Normal RU patterns have been described, using objective ROI placement methods, for the lateral view of the stifle in scintigrams acquired at one veterinary hospital [10]. These patterns were bilaterally symmetrical, repeatable and not altered by forelimb lameness. Normal stifle RU patterns have also been described subjectively in Standardbreds, Thoroughbreds and sport horses [9,11,12] and it has been shown that quantitative evaluation of stifle scintigraphy in horses cannot replace the visual subjective examination of the scintigram [13]. A standardized method of positioning the lateral view should improve the accuracy of applying these patterns to scintigrams, and it should reduce the variations introduced by different operators and equipment.

The aims of this study were to develop a reliable method to obtain a lateral scintigram of the equine stifle based on fixed anatomical landmarks (point sources guided method) and measure the resulting foot to gamma camera angle (FC angle) on sequential measurements of the same horse and of different horses. The hypotheses were; the point sources guided method would result in a consistent FC angle for the lateral scintigram when horses were measured repeatedly, and that there would be low variation of the FC angle between horses.

## Methods

### Study group

During February to June 2011, client-owned horses presented for scintigraphic investigation of poor performance, lameness or dental disease were selected (Table 1). None of the horses had lameness specifically or solely localized to the stifles. For each horse one stifle was chosen, either in a random way if the clinical symptoms were not lateralized, or in the contra-lateral limb of the clinically symptomatic hind leg. In total, 13 left and 9 right stifle joints were evaluated. The project had ethical approval and the owner's consent was obtained for each horse. Twenty-two horses; 17 Warmbloods, 3 riding ponies, one Thoroughbred and one Standardbred aging from 4 to 15 years were included. There were 12 mares, 6 stallions and 4 geldings.

**Table 1:** Indications for nuclear scintigraphic examination in 22 horses

Poor performance	9
Lame with equivocal results of diagnostic analgesia	4
Lame after known trauma	3
Refuse to be ridden	2
Dental disease	2
Lame with localized site of pain	2

### Identification of anatomical landmarks

Using ultrasound guidance (Acuson Antares; VFX 13–5 MHz Linear Probe, Siemens Medical Solutions, USA), the origins of the medial collateral ligament (MCL) and the lateral collateral ligament (LCL) were identified. The area with overlap in a lateral to medial plane of the caudal half of the origin of the LCL and the cranial half of the origin of the MCL was used to define a true lateral view of the stifle [14]. Correction fluid (Tipp-Ex Rapid Correction Fluid Foam Bic, France) markings were made on the skin as close as possible to the identified anatomical structures. All ultrasounds were performed before the scintigraphic examination by the same operator (MGM).

**Figure 1:**
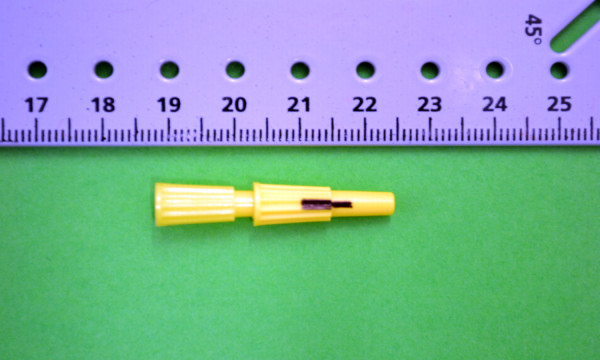
**Radioactive point sources. **These were small sealed plastic capsules (7 mm in length x 2,5 mm in diameter) containing ^99m^Tc-HDP in saline that were glued to the horses skin. On the photos, the area containing the radioactivity is represented by the black line drawn on the capsule.

Radioactive point sources were glued (Karlssons Universalklister UHU Nordic, Germany) on the correction fluid dots. These point sources consisted of small sealed plastic capsules (7 mm in length x 2,5 mm in diameter) containing ^99m^Tc-HDP (Figure 1). The medial capsule was filled with approximately 30 MBq (810 μCi) of ^99m^Tc-HDP diluted in saline to make a total volume of 0.1 mL, and the lateral capsule contained approximately 1 MBq (27 μCi) of ^99m^Tc-HDP diluted in saline to make a total volume of 0.02 mL. To avoid overlapping of the gamma rays from the point sources, one was glued slightly distal to the other, but always in the same vertical plane as the correction fluid dot.

**Figure 2:**
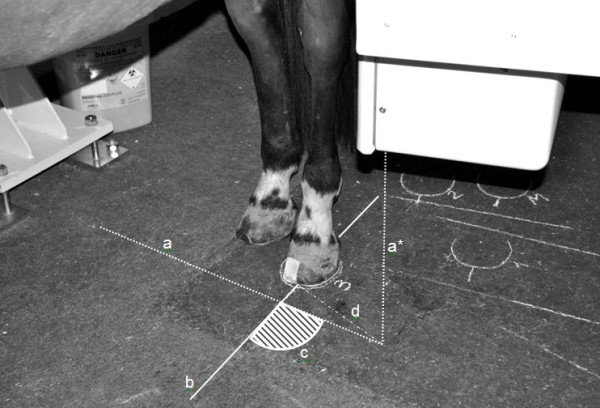
**Schematic representation of the foot-camera (FC) angle measurement. **The position of the vertical axis of the gamma camera was projected using a manual laser level device placed on the center of the face of the gamma camera and producing a fan beam of light perpendicular to the ground (dotted line (a*)). The projected axis was marked on the floor with chalk (dotted line (a)).The mid-sagittal foot-axis is indicated by continuous line (b). To calculate the FC angle (c), the Law of Cosines was applied. Three distances were measured: the distance from the foot and gamma camera axes intersection to the tip of the foot (portion of the continuous line (b)), the distance from the foot and gamma camera axes intersection to the point vertically below the center of the gamma camera face (portion of the dotted line (a)); and the distance from the tip of the foot to the point vertically below the center of the gamma camera face (dashed and dotted line (d)).

In order to be able to measure the angle between the mid-sagittal axis of the foot and the gamma camera-axis (FC angle), measurement points and lines were defined (Figure 2). The bisecting line of the frog, passing through the central sulcus was defined on an uplifted foot and the mid-point of the bulbs of the heel was marked on the foot. The location of the bisecting line on the tip of the toe of the hoof was identified by the lateral margin of tape adhered to the hoof.

### Scintigraphic image acquisition

The images included in the study were taken following the completion of the scintigraphic examination done to investigate the presenting clinical problem. Each horse was injected with ^99m^Technetium-hydroxymethylene diphosphonate (^99m^Tc-HDP) intra-venous (IV) (5 GBq/horse; 135 mCi/horse) in the left or right jugular vein, and images were acquired 6–7 hours after the injection. The horses were initially sedated with IV detomidine hydrochloride (Cepesedan™ 10 mg/mL, Scanvet, Sweden) (20-40 μg/kg of body weight) in combination with butorphanol (Butador VET™ 10 mg/mL, Vetoquinol, Sweden) (0.1 mg/kg of body weight). If necessary, repeated boluses of detomidine hydrochloride were given IV (10-30 μg/kg of body weight). The images were obtained using a low-energy, general-purpose collimator and a 58 cm by 40 cm rectangular field of view GE gamma camera (Gamma Camera Millenium. General Electrics Medical Systems, USA) linked to a dedicated nuclear medicine computer system (Hermes Gold3 Browser V.3.8. Nuclear Diagnostics AB, Stockholm, Sweden). All images were acquired dynamically as a series of 90 1 s frames, using a 256 x 256 matrix. For all images, motion correction software was used to align each frame to produce a static image. All images were evaluated using a commercial software package (Hermes Gold3 Browser V.3.8. Nuclear Diagnostics AB, Stockholm, Sweden).

### Positioning and measurement procedure

**Figure 3:**
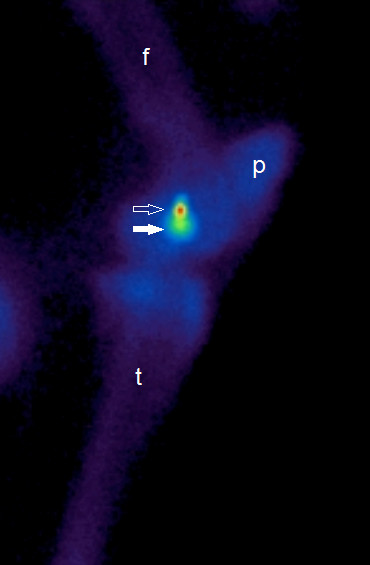
**A lateral stifle scintigram showing vertical alignment of radioactive point sources. **Due to increased distance to the gamma camera, the definition of medial point source (arrow) was poorer than the lateral point source (white arrow). In this image, the lateral point source is proximal to the medial one. Femur (f), patella (p) and tibia (t).

For all acquisitions the horses were evenly weight bearing and standing squarely on all limbs. The gamma camera was positioned as close as possible to the stifle, and the face of the camera was perpendicular to the floor. A lateral scintigram of the stifle was defined as the image showing the two radioactive point sources aligned in the same vertical plane. The alignment of the point sources was assessed subjectively by two operators (MGM, MB) on the live image, and the position of the gamma camera was adjusted so that the two points appeared vertically aligned (Figure 3). When there was agreement between the two operators that the points were considered vertically aligned, the image acquisition was started.

With the horse weight bearing and in position, a line was drawn on the floor around the contours of the hoof and the tip of the toe of the hoof and the mid-point of the bulbs of the heels were marked on this line. Later a line indicating the mid-sagittal axis of the foot was drawn between the tip of the toe and mid-point of the bulbs of the heel. The position of the vertical axis of the gamma camera was projected using a laser level device (Black and Decker BDL 120. Black and Decker, Sweden) placed on the center of the face of the gamma camera which produced a fan beam of light perpendicular to the ground and thus created a line of light on the floor. This line of light was drawn on the floor with chalk.

The FC angle was calculated using the Law of Cosines from chalk lines drawn on the floor during image acquisition. The Law of Cosines was used as it allows calculation of any angle of a triangle when two of the angles or lengths of the sides are known. As a chalk line is a few millimeters thick, the measurements of the distances were taken in the middle of the drawn lines. Between each acquisition the horse was walked several steps away from the gamma camera while the camera remained locked in position when lines were completed and measurements of the FC angles were performed. This acquisition and measurement procedure were repeated at least five times for each horse.

### Post-acquisition image evaluation

After the image acquisitions and motion correction processing, lateral images were reviewed using a continuous blue-green-red color scale and vertical line profiles. For images and the corresponding measured FC angles to be included in the study the medial and lateral radioactive point sources had to be aligned within in a 5 pixels wide vertical linear region (total width = 7.8 mm) that had vertical line profiles as its cranial and caudal boundaries. The vertical region was first centered on the point of maximal intensity of the lateral point source, then the position of the point of maximal intensity of the medial point source was evaluated. For the radioactive point sources to be considered vertically aligned at least the point of maximal activity of the medial point source had to be within this vertical plane. If there were more than five lateral scintigrams of a horse that satisfied the inclusion criteria, then the first five acquisitions were used. This resulted in five repeated measurements of the FC angle for each horse.

### Statistics

Descriptive statistics were calculated for the FC angles. Coefficients of variation (COV) were calculated for the mean of the means of each group of acquisitions one to five for all horses and for the mean of the means of the FC angle on each horse.

All statistical analyses were performed using a statistical software package (Minitab 15. Minitab Inc. State college, Pennsylvania, USA).

## Results

Post-acquisition image evaluation of 112 images was required to give five images per horse. With 22 horses enrolled, only two of the images subjectively positioned under the point source guidance were rejected due to obliquity using the post-acquisition image evaluation criteria.

**Figure 4:**
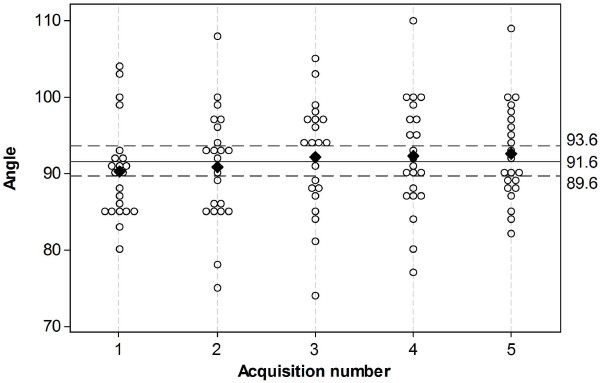
**Plot representing the distribution of the FC angle value per acquisition (n = 5) in each individual horse (n = 22). **Unfilled circles represent the values of the FC angles. For equal values, the circles are offset and positioned side by side. The mean value for each measurement is represented by black diamond. The mean of these means (91.6°) is represented by a solid horizontal line, the maximal and minimal limits of the 95 % confidence interval by dashed horizontal lines. FC angle = Foot Camera angle.

FC angles for all horses grouped together as acquisition one to five, are shown in Figure 4. The mean FC angle for each acquisition group varied between 90.2° and 92.2° with a mean of the means of 91.6 ± 2° (2 SD) and a COV of 1.1%.

**Figure 5:**
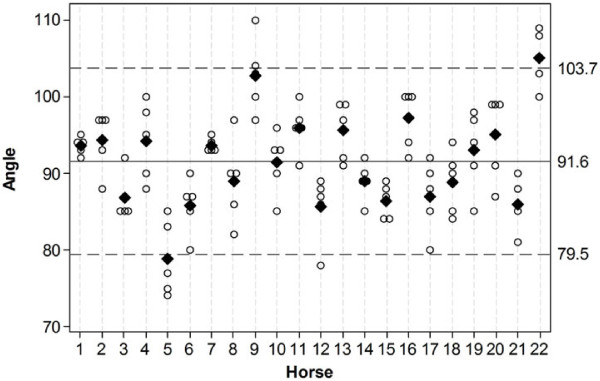
**Plot representing the distribution of the five foot camera (FC) angle values obtained in each individual horse (n = 22). **Unfilled circles represent the values of the angles. For equal values, the circles are offset and positioned side by side. A diamond represents the mean value for each horse. The mean of the mean angles (91.6°) is represented by a solid horizontal line, the maximal and minimal limits of the 95 % CI (confidence interval) by dashed horizontal lines.

FC angles for each of the five repeated measurements are shown per horse in Figure 5*.* The mean FC angle for each individual horse from the five repeated measurements ranged from 78.8° to 105°. The mean of the mean FC angles for the 22 horses was 91.6 ± 12.1° (2 SD) and a COV of 6.6%.

## Discussion

The low COV (1.1%) for repeated FC angle acquisitions using the point sources guided method supports our hypothesis that a method based on landmarks positioned on anatomical structures of the stifle should result in a consistent FC angle for lateral scintigrams of the same horse. Furthermore, the results imply that the technique is a suitable method to use as reference standard for lateral scintigrams of equine stifles.

Based on anatomical specimens and recently published work on the radiographic anatomy of the equine stifle soft tissue attachments [14], the area where the caudal half of the origin of the LCL overlaps with the cranial half of the origin of MCL was used to define a lateral view of the stifle. These anatomical landmarks were chosen because; only minimal variation of the positions of these landmarks was expected between horses, the landmarks were close to the center of the stifle region, the superficial location of the landmarks allowed fast and easy identification by ultrasound, and the capsules could be fixed very close to these structures.

The five repeated measurements of the FC angles were done both in an attempt to minimize random error in the alignment of a true lateral scintigram and to investigate the amount of variation that occurs when repositioning the horse. The alignment of the point sources was assessed subjectively before the acquisition was started and these images were later evaluated using a vertical linear region to confirm vertical alignment. The vertical region was five pixels wide, which is 7.8 mm wide for the field of view and image matrix size used. Considering the point spread function from a point source of gamma-rays [15], we aimed to include at least the central half of the medial point source activity within the profile limits as this region of higher count density was likely to represent the medial capsule location. The subjective alignment of the lateral view done using the point sources resulted in 98.2% of the images being correctly aligned when the more rigorous post-acquisition evaluation was performed. This indicates that it is reasonable to rely only on the subjective assessment of point sources position of the live image to decide the correct positioning of the gamma camera relative to the stifle for the lateral scintigram.

The use of visual anatomical landmarks is a common method for positioning of equine scintigrams [10]. The relatively large size and rounded shape of the equine stifle region means that it lacks well defined visual anatomical landmarks that can be used for positioning of lateral views. Instead, adjacent anatomical regions such as the distal limb and the body can be used to guide the position of the gamma camera [16]. A feature that can be well defined in almost all horses is the mid-sagittal axis of the foot. Using this as a reference line and knowing the correct FC angle relative to this, would be a simple and practical method to positioning the camera for the lateral view. The aim of this study was to establish a method to obtain consistent lateral stifle scintigrams. Repeatable positions of the horse and the gamma camera are important to get images that have similar spatial relationships of the stifle anatomy. The view angle must be correct at the time of acquisition since post-processing software cannot compensate for positioning differences. The acceptable degree of variation of view angle for a scintigram, and how this affects the diagnostic information in the image is unknown and requires further investigation.

The radioactive capsule guided lateral scintigrams of the stifle resulted in a mean FC angle of 91.6°, a COV value of 6.6%, and the 95% CI for the FC angle varied 24.2°. This implies that if the mid-sagittal axis of the foot is used to guide gamma camera positioning for lateral stifle scintigrams an FC angle of approximately 92° could be used, but it should be expected that there will be mild to moderate variation in the relative position of uptake from structures in the resulting scintigram. Thus, the hypothesis that there would be low variation of FC angle between horses is partially rejected, and so if a straight lateral view is important for image interpretation then use of the capsule guided method is recommended. However, measurement of FC angles for individual horses should be very useful for sequential and follow-up image acquisitions. Positioning guided by the FC angle in the same individual should result in very low stifle scintigram view angle variation between examinations and thus minimize changes in the spatial relationships of RUs in the scintigrams. This should help to maximize diagnostic accuracy for follow-up examinations of stifle pathology.

The collateral ligaments and the conformation of the equine hind limb joints from the stifle to the distal interphalangeal joints permit little rotation [17,18], thus the spatial relationship between the stifle and the foot in an individual is likely to remain constant. This is supported by the low COV for the mean values of the five repeated measurements (1.1%), which indicates that the major source of variation is the horse rather than the method used to position the lateral view, and that the spatial relationship between the stifle and foot is stable in individual horses. The most likely explanation for the higher variation in FC angle between horses (COV 6.6%) is anatomical variations in shape, angle and rotation of the distal limb relative to the stifle. There were several horse breeds included in this study and it is possible that anatomical variation between breeds will be greater than the variation within breeds. To further investigate the use of the sagittal foot axis as an anatomical landmark to position the lateral scintigram, it might be useful to study horses of one specific breed since variation within a breed might be less than the variation in the entire horse population. In the horse with the minimal mean FC angle of this population (horse 5), three of the five FC angles were below values of other horses. This horse was the only Thoroughbred in the study, so a breed related difference in conformation should be considered as a possible cause. Two of the study horses (horse 9 and 22), which were both Warmbloods had angles higher than the other horses (Figure 5). Since 17 of 22 horses in the study were Warmbloods a specific breed related conformation variation is not a convincing explanation for these higher values.

The skin surface location of the phantom capsules relative to the femur could be considered a limitation of the point sources guided method. However, in all horses, the soft tissue thickness separating the skin to the bone surface at the ligaments origins site did not exceed 2 cm. With the distances and angles used parallax error is considered unlikely to have influenced the results.

The consistent positioning produced using the point sources guided method makes it useful in a clinical situation. The method can be used to achieve consistent view angles for the lateral scintigram and the method is likely to be very stable when used by different operators. This should result in lateral stifle scintigrams that can be more accurately compared between and within individual horses. A reliable scintigraphic view should maximize the accuracy of subjective and objective scintigram evaluation, which is particularly important when semi-qualitative methods are used since comparative ratios between different ROIs are only valid if anatomical placement of the ROIs is precise. A similar situation exists with the equine sacroiliac joints, where the anatomic location of the sacroiliac joints using a phantom model in the dorsal view needed to be confirmed before consistent analyses of scintigrams of this region could be done [8].

The point source guided method can also help to reduce the radiation dose received by the staff during scintigraphic examinations, since accurate camera positioning should be quicker, and repeat acquisitions due to oblique angles of view should be less frequent. A disadvantage of the method is the need to prepare radioactive point sources in advance and the time spent to identify the anatomical landmarks. When the method is used in a clinical situation the capsules should be used to achieve the correct angle of view, but it is very important that the capsules are removed prior to image acquisition. If the capsules are left in place then significant count stealing will occur and this will result in a major reduction in diagnostic quality of the scintigram.

## Conclusions

The point source guided positioning method demonstrated low variability to obtain repeated lateral stifle scintigrams in horses of variable size, age and clinical status. The method was felt to be easy and required only standard imaging equipment. We consider this method suitable as a reference standard method to obtain lateral scintigrams of the equine stifle, and that it will be of value in clinical scintigraphy and in research. The use of an FC angle to position the gamma camera would be expected to result in a higher variation in the positioning of the lateral stifle scintigram compared to the radioactive capsule guided method. The use of alignment of specifically located radioactive capsules may also be applied in other regions to standardize scintigraphic views.

## Abbrevations

FC angle: angle between the mid-sagittal foot-axis and the vertical axis of the gamma camera; RU: radiopharmaceutical uptake; MCL: medial collateral ligament; LCL: lateral collateral ligament.

## Competing of interests

The authors declare that they have no competing interests.

## Authors’ contributions

MGM has been involved in the initial design of the study and has been main responsible for data acquisition and analysis and drafting the manuscript, CL has been involved in the initial design of the study, performed the statistical analysis and participated in the writing of the manuscript, MB has been involved in the initial design of the study and has been main responsible for the data acquisition, KH was involved in the development of the study and contributed to the writing of the manuscript. All authors have read and approved the final manuscript.
